# Everybody's Page

**Published:** 1890-04-12

**Authors:** 


### Everybody's Page.

Sir Lyon Playfair points out that, concurrently with an increase of tuberculosis amongst cattle, consumption of one kind or another increases amongst children.

Far away from the influence of civilized man gesture language is still extant in Australia. Some of the tribes possess such an excellent code that it is almost as efficient as a spoken language. If there are any unfortunate enough to be deaf and dumb among these Aborigines, they should feel their absence of physical perfection comparatively slightly.

The stature of dwellers on the coasts, according to Mr. Gattie, in this month's *Fortnightly*, is greater than that of men of the same descent living inland, while a mountainous district and a vigorous climate tend, whilst expanding the chest, to stunt the growth, and thus produce sturdy, thick-set specimens of humanity. Growth is also affected adversely by damp air and fen.

The French Anti-Tobacco Society is about to open a new competition, and it will give two prizes, one for a paper on the "Influence of Tobacco and Nicotine on the Digestive Functions," and another to the French or foreign doctor who will relate the greatest number of recoveries from nicotine affections through the agency of hypnotism by the giving up of the use of tobacco.

It is not every prison which can boast of supporting itself entirely, and it would be a good thing if there were many more in a similar position. The Detroit Prison, Michigan, is a chair manufactory, all the prisoners being employed in this industry, and the manager is able to pay all salaries and expenses of the prison out of the proceeds, and also to hand over a large sum to the City authorities.

Success seems veritably to have crowned the efforts of the district visitors in Kensington who have taken up the inspection of sanitary matters in that parish. Rich folk can afford to look after their own affairs, but the poor in the majority of cases have neither the means, nor the time, nor the knowledge necessary to set the sanitary arrangements of their dwellings in order ; and after all, when the rich man fights the poor man's battle he is doing himself a turn at the same time, for he is waging war against the germs of disease, which knock at the door of the rich and the poor alike.

The man whose idea it was to make the tops of the omnibusses comfortable and get-at-able, had not only a keen eye to business, but he was a benefactor in good earnest to his fellow-men. It is just days like these last sunny ones of March that show how garden seats are appreciated ; and when we see the Hospital nurses and the tired workwomen and all who need fresh air thoroughly enjoying their pennyworth upstairs, we feel there is no creature in the world more deserving of pity than the genteel female who still retains the idea that Mrs. Grundy will consider her bold if she mounts the steps.

The excessive wear and tear to which salt-pans are subjected in the manufacture of salt have, for a long while, taxed the ingenuity of inventors to overcome. An Austrian seems at last to have met with success in this direction, and the method of manufacturing salt which has been carried on without change ever since that commodity was first made will now probably be revolutionised. When so many of our national industries have undergone such radical changes in the course of the past fifty years, it is curious that no advance should have been made during so many centuries in a trade of such importance. The change is the more important in that it promises not only to benefit manufacturer and consumer, but to resuscitate in this country a declining industry.

Snow collected during its fall contains living bacteria in considerable numbers. The bacteria originate partly in aqueous vapours, which are transformed into snow, but chiefly in the air ; they are, in fact, carried away by the snowflakes during their passage through the atmosphere.

Mr. A. P. Laurie claims that he has solved the difficulty of preventing paints used by artists from fading. His endeavours have been made in the direction of obtaining a medium which is unaffected by moisture. He has made an amber varnish out of sulphate of copper which, after drying, remains perfectly colourless.

The *Journal d'Hygiène* gives much praise to M. Féret for his invention of an excellent table for the use of children at school, which can be most simply raised or lowered to suit each child's height ; it prevents the pupil leaning up against the desk and so stopping the expansion of his chest, and the eyesight is also preserved. Parents and guardians please take this to heart.

Miss Clementina Black's criticism on marriage comes like a calm after the storm and chaos into which Mrs. Mona Caird threatened to plunge our social system. We think that Miss Black strikes the key-note of the misery of many of the wretched marriages in the world when she says that in marriage, as in every other great question in life, "to enter rashly upon serious undertakings is to invite ruin," and again, "No opportunity and no form of marriage that can be devised can make beautiful or civilised the relations of those who are themselves unbeautiful or uncivilised.

Scotch people are proverbially supposed to be knowing, yet report says that many of the inhabitants of Glasgow were the victims of a plot some years ago. A chemist advertised all over the hoardings and in every available spot in the town, "Certain Death to Fleas." Now fleas may be very vulgar to mention, but they are also annoying, and the Glasgow folk in all good faith bought largely of the "Certain Death." They received a neat little box labelled, "Full directions inside ; and the directions were : "When caught, a pinch of the powder put on to the flea will cause instantaneous death."

Hypnotism is making vast strides as an anæsthetic, and the account in the *British Medical Journal* of the recent demonstration of its power at Messrs. Carters' at Leeds makes us feel quite cheerful about our future visits to the dentist. The prospect of bidding adieu to the awful wrench or the horrid alternative of our old friend "the gas" is most comforting ; instead, we shall be sent into a calm, refreshing sleep, and we shall awaken (like Dr. Milne Bramwell's patient, who had sixteen stumps removed and awoke smiling !) as lively as a cricket, and as if nothing had happened. Blessed hypnotism !

"The dormouse is a biting and angry beast, and therefore seldom taken alive." This is one of the many gems of Natural History found in the "Druggist's Shop Opened." Dormice must have marched with the times since 1693, and become more civilised. They certainly have a better time in one respect now than formerly, for they were considered a great delicacy, and the luxurious Romans used to eat as many of them as they could get. "The flesh is good against pneumonia," the fat against deafness, and the ashes of the whole beast blown into the eyes, take away spots and films, and wonderfully clear the sight." This is valuable information, for up to now we have ignorantly believed that ashes in the eyes had anything but a salutary effect.

